# Australia’s Ongoing Legacy of Asbestos: Significant Challenges Remain Even after the Complete Banning of Asbestos Almost Fifteen Years Ago

**DOI:** 10.3390/ijerph15020384

**Published:** 2018-02-23

**Authors:** Matthew Soeberg, Deborah A. Vallance, Victoria Keena, Ken Takahashi, James Leigh

**Affiliations:** 1 Asbestos Diseases Research Institute, P.O. Box 3628, Rhodes, NSW 2138, Australia; victoria.keena@sydney.edu.au (V.K.); ken.takahashi@sydney.edu.au (K.T.); jleigh@bigpond.com (J.L.); 2 Australian Manufacturing Workers’ Union, P.O. Box 160, Granville, NSW 2142, Australia; Deb.Vallance@amwu.org.au

**Keywords:** asbestos, Australia, prevention, mesothelioma, ban, asbestosis

## Abstract

The most effective way of reducing the global burden of asbestos-related diseases is through the implementation of asbestos bans and minimising occupational and non-occupational exposure to respirable asbestos fibres. Australia’s asbestos consumption peaked in the 1970s with Australia widely thought to have had among the highest per-capita asbestos consumption level of any country. Australia’s discontinuation of all forms of asbestos and asbestos-containing products and materials did not occur at a single point of time. Crocidolite consumption ceased in the late 1960s, followed by amosite consumption stopping in the mid 1980s. Despite significant government reports being published in 1990 and 1999, it was not until the end of 2003 that a complete ban on all forms of asbestos (crocidolite, amosite, and chrysotile) was introduced in Australia. The sustained efforts of trade unions and non-governmental organisations were essential in forcing the Australian government to finally implement the 2003 asbestos ban. Trade unions and non-government organisations continue to play a key role today in monitoring the government’s response to Australian asbestos-related disease epidemic. There are significant challenges that remain in Australia, despite a complete asbestos ban being implemented almost fifteen years ago. The Australian epidemic of asbestos-related disease has only now reached its peak. A total of 16,679 people were newly diagnosed with malignant mesothelioma between 1982 and 2016, with 84% of cases occurring in men. There has been a stabilisation of the age-standardised malignant mesothelioma incidence rate in the last 10 years. In 2016, the incidence rate per 100,000 was 2.5 using the Australian standard population and 1.3 using the Segi world standard population. Despite Australia’s complete asbestos ban being in place since 2003, public health efforts must continue to focus on preventing the devastating effects of avoidable asbestos-related diseases, including occupational and non-occupational groups who are potentially at risk from exposure to respirable asbestos fibres.

## 1. Introduction

Almost fifteen years after a complete ban on asbestos was implemented in Australia, protecting Australians from the harms of asbestos is increasingly recognised as a complex public policy problem. As recently as November 2017, a Senate inquiry into protecting Australians from the threat of asbestos in non-conforming building products [1] stated that:

“Asbestos safety is a complex policy and operational area that requires coordinated efforts on a national scale. As such, a number of Commonwealth, state and territory government agencies have responsibilities for monitoring asbestos across a range of areas including; workplace safety, border protection, environmental protection, public health and consumer safety.”

(p. 14, Interim Report of the Economics References Committee, the Australian Senate)

Australia is often looked to as a country that has among the highest incidence rates of malignant mesothelioma due to high per-capita asbestos exposure in the past [2,3]. As early as the late 1980s, epidemiological researchers started to investigate the emerging issue in Australia of asbestos-related disease, with a focus on malignant mesothelioma [4,5,6]. Moving forward forty years later, public health researchers and advocates still have a key role to play in measuring the pattern of Australia’s asbestos-related disease epidemic curve and understanding the risks of in situ asbestos and asbestos containing materials [7,8,9,10,11,12,13,14].

In this article, we describe how asbestos was used in Australia and the various events related to the time when asbestos was banned. We also reflect on the key campaigns from the manufacturing unions that led to asbestos ban policy changes, particularly the complete ban of asbestos in 2003. More recent developments, such as the establishment of the world’s first national asbestos safety and eradication agency and the re-establishment of the national mesothelioma surveillance system, are highlighted. Finally, we describe patterns of asbestos-related disease in Australia and how the peak of disease is only now being observed. We present both malignant and non-malignant asbestos-related disease data.

The International Agency for Research Cancer has stated that asbestos causes malignant mesothelioma and cancer of the lung, larynx and ovary [15].

Here, we describe patterns over time in malignant mesothelioma incidence as well as mortality and hospitalisation data for asbestosis and pleural plaques. Global burden of disease data for occupational carcinogens, including data for Australia, are published elsewhere [16] and include estimates for other asbestos-related cancers. Briefly, recent Global Burden of Disease data from the Institute of Health Metrics and Evaluation (https://vizhub.healthdata.org/gbd-compare/) estimate that a total of 4048 asbestos-related deaths due to occupational asbestos exposure occurred in Australia in 2016 with 75% of those deaths being due to asbestos-related lung cancer (*n* = 3017) and 19% being due to malignant mesothelioma (*n* = 766). The remaining six per cent of deaths were due to larynx cancer, ovarian cancer or asbestosis (*n* = 48, 140, and 77, respectively). In this article, we have primarily relied on national administrative health datasets including cancer registries, mortality and hospitalisation data to accurately describe Australia’s asbestos-related disease patterns. Other data are available. However, these data are unlikely to capture the total burden of asbestos-related disease. For example, workers’ compensation data collection nationally in Australia only provide information about successful malignant mesothelioma and asbestosis compensation claims. A limitation of interpreting these data to more comprehensively understand the recent burden of asbestos-related diseases in Australia is that the number of workers’ compensation claims do not match the number of asbestos-related deaths. National data show that only 33 people received workers’ compensation for asbestosis during 2014–2015 compared to the 127 who are known to have died from asbestosis in 2015.

## 2. Use and Discontinuation of Asbestos in Australia

Australia is a country where high levels of raw asbestos and asbestos-containing products and materials were consumed. This has resulted in adverse health, social and economic outcomes for the Australian community with these effects still being observed today.

Australia had the world’s highest per-capita asbestos consumption rate in the 1950s, primarily from mining, the use of asbestos in construction materials, and also the importation of asbestos-containing products [3,17]. Asbestos consumption in Australia peaked in the 1970s at 704,425 metric tonnes (1970–1979 aggregate) [18]. A total of 82 tonnes of asbestos was mined between 1880 and 1889 made up of 47 tonnes of amphiboles at Jones’ Creek, near Gundagai, New South Wales, and 35 tonnes of chrysotile at Anderson’s Creek, Tasmania.

There was a gradual increase of asbestos production in Australia from the late 1880s up until the late 1930s, mainly from chrysotile deposits. A transition occurred in the late 1930s when mining commenced at Wittenoom, Western Australia, in 1937. The main asbestos produced from mining at Wittenoom was predominately crocidolite. The Wittenoom mine closed in 1966. The closure of the Wittenoom asbestos mine was for economic reasons such as transportation and labour costs, rather than for controlling the occurrence of dust diseases [19].

The Australian state to mine the largest tonnage of chrysotile was New South Wales. There were two main asbestos mines in New South Wales. Mining of asbestos in Baryulgil, New South Wales, started in 1944 and operated for several decades, finally closing in 1979. Chrysotile was also mined at Woodsreef, New South Wales, from the early 1970s through to 1983. Following the closure of the Wittenoom mine, around 122 tonnes of crocidolite was mined in South Australia.

Australian crocidolite and chrysotile asbestos fibres were exported to a number of countries including Japan, the United Kingdom and the United States. The United States and European region in particular imported large amounts of Australian crocidolite. Many workers were exposed to asbestos through the process of exporting crocidolite and chrysotile to other countries. From the late 1960s, there was a decline in exportation of Australian asbestos.

In the late 1970s, the Australian Broadcasting Corporation produced a series of radio programs focusing on the effects of asbestos among workers in the industry. This publicity around the effect of asbestos on workers led to increased awareness of the Baryulgil chrysotile mine and its effect on the local Aboriginal populations. In 1984, a Parliamentary Inquiry was held to investigate (1) the impacts of asbestos exposure for Aboriginal people who lived and or worked at Baryulgil (2) the protection and promotion of health and welfare for Aboriginal people impacted by the Baryulgil mining operations, and (3) compensation provisions available to people adversely affected by Baryulgil mining and processing activities [20]. The social and health effects of chrysotile mining at Baryulgil are still coming to light.

Naturally occurring chrysotile deposits at Woodsreef, New South Wales, began to be mined in 1971. The mine was open-cast with dry milling. The Woodsreef mine ceased production of chrysotile in 1983 due to difficulty in complying with Australian dust control regulations as well as the global drop in demand for asbestos production occurring at the same time. To the authors’ knowledge, there have been no epidemiological cancer or mortality follow-up studies of the Woodsreef mine workers. This is a substantial gap in the story of Australia’s asbestos-related disease epidemic.

Australia imported raw asbestos from Canada (chrysotile) and South Africa (crocidolite and amosite). About two times the volume of chrysotile was imported from overseas than was produced in Australia. However, the majority of crocidolite use in Australia was from local production rather than importation. Australia also imported many manufactured asbestos-containing products from Germany, Japan, the United Kingdom and the United States, with the first importations thought to have started in 1929. These products included: asbestos-containing friction material and gaskets; asbestos yard, cord and fabric; asbestos joint and millboard; and asbestos-containing cement. The asbestos cement manufacturing industry was one of the largest users of asbestos fibre. In Australia, almost all (90%) of asbestos fibre consumption occurred in the asbestos cement manufacturing industry.

Chrysotile use continued in friction products, sealing gaskets and adhesives until 2003, with a complete ban on all importation and use of asbestos across Australia occurring in 2003 [18,21]. Many asbestos-containing products and materials remain in situ in Australia’s residential and non-residential buildings, as well as in water and sewerage piping.

## 3. Campaigning for the Banning of Asbestos in Australia up to the Complete Ban of Asbestos in 2003

The end of 2003 marked an important milestone in Australia’s asbestos consumption story. From the end of 2003, with a few time-limited exceptions, asbestos and asbestos-containing products could no longer be imported into Australia. The time-limited exemptions related to use by the Australian Defence Organisation for chrysotile in products or parts where the parts were mission-critical and there was no reasonable alternative to the use of chrysotile. The exemptions included: compressed asbestos fibre gaskets used with saturated steam or superheated; compressed asbestos fibre gaskets used with chlorine in liquid chlorine service plants; and diaphragms for use in electrolytic cells.

The trade union movement played a critical role in prompting State and Commonwealth agencies to act in the best interest of the Australian community in terms of protecting health and employment. The 2003 date was eight years after the deadline for a phased-out asbestos ban that a 1992 National Conference of the Australian Manufacturing Workers Union had endorsed [22], with a phased-out approach considered to assist in protecting employment opportunities for workers handling asbestos-containing products and materials as well as protecting all workers from the harmful health effects of asbestos exposure.

One of the most important trade unions in Australia’s progress towards a complete asbestos ban was the Australian Manufacturing Workers’ Union (AMWU). A large member base of the AMWU was maintenance and tradespeople who were exposed to asbestos lagging and friction products. Automotive, power and shipbuilding industry workers were also likely to be significantly exposed to asbestos through Australia’s high levels of manufacturing and use of asbestos-containing brakes, clutches and gaskets. In particular, these asbestos-containing products were used in the automotive industry. The challenge for the AMWU in protecting its members was twofold. On one hand, many members were suffering the devastating, and often fatal, effects of exposure to asbestos in their workplace. On the other hand, there was also a group of workers whose employment relied on the manufacturing as well as installation or maintenance of asbestos-containing products. Protection of employment among members was critical given the high unemployment rates that had plagued Australia during the 1980s and 1990s.

A phase-out approach to the importation of all raw asbestos was first recommended in Australia in 1990 through a Victorian Government inquiry on asbestos use [23]. At the time, there was a large demand for the replacement of asbestos-containing vehicle brakes. It was considered by the replacement brake market that non-asbestos replacement brakes were not as safe and had suboptimal performance compared to asbestos-containing replacement brakes. The Victorian Government’s report into asbestos use recommended that “where a vehicle braking system was designed and built with non-asbestos friction materials, only equivalent standard non-asbestos materials may be used in subsequent repair and maintenance”. However, this issue was not heeded by governments that implemented the inquiries recommendations. Further, claims made by the asbestos industry at the time of the 1990 inquiry that chrysotile consumption would reduce in Australia did not materialise.

The introduction in Victoria, Australia, of the Occupational Health and Safety (Asbestos). Regulations 1992 marked a significant shift in the protection of Australian workers from asbestos exposure. The regulations were established to prevent asbestos-related diseases for workers exposed to airborne asbestos in occupational settings. The trade union movement played a key role in advocating for the regulations to be developed and implemented. The regulations specified a number of measurable asbestos exposure standards over a minimum period of four hours including 0.1 fibre/millilitre for removal of asbestos from any building, structure or shop, 0.1 fibre/millilitre for all forms of asbestos other than chrysotile, 0.5 fibre/millilitre for chrysotile, and 0.1 fibre/millilitre for unknown asbestos types. There was no provision in the regulations relating to the production of asbestos products in the state. Importantly, these regulations only related to the state of Victoria and did not apply at the national level.

A shift in focus from the state to national level occurred in the mid 1990s. The Australian Government’s National Industrial Chemicals Notification and Scheme started to investigate occupational exposure to chrysotile through its Priority Existing Chemicals (PEC) program [24]. Discussions on lowering the chrysotile asbestos exposure standard at the national level stalled. However, this provided an opportunity for trade unions and non-government organisations to gather evidence on chrysotile asbestos in various employment sectors. Also during the 1990s, importation of asbestos and asbestos-containing products and materials continued with ongoing claims from automotive manufacturers that non-asbestos friction products were substandard which would lead to risks for Australian motorists. Further, a common concern for both the AMWU and manufacturers was the protection of employment opportunities for workers.

In 1999, the National Industrial Chemicals Notification and Scheme released its full public report on chrysotile asbestos [24]. Three of the eight key recommendations outlined in the report include: (a) that a phase-out of chrysotile be implemented in Australia; (b) that consideration be given by the National Occupational Health and Safety Commission (NOHSC) to the national exposure standard for chrysotile; and (c) that manufacturers, suppliers and users comply with the hazardous substance model regulations developed by the NOHSC. The Australian exposure standard for chrysotile in 1998 was 1.0 fibre/millilitre and 0.1 fibre/millilitre for exposure to all other asbestos fibres [24]. More recently, the Australian exposure standard for exposure to all asbestos fibre types is 0.1 fibre/millilitre [25].

Despite the 1999 PEC report, there appeared to be little action taken by the NOHSC to implement the PEC recommendations, including setting an asbestos ban timeframe. Sustained efforts of trade unions and non-government organisations in advocating to the state and national governments for the protection of workers from the effects of asbestos and asbestos-containing products and materials reached a peak in 2000. There was also a need to act quickly because of the concerns from various trade unions about the labour market impacts of abruptly stopping the supply of asbestos and asbestos-containing products. A roundtable meeting brought together unions, industry and the Victorian state government. By the end of 2000, a roadmap for phasing out the importation of raw asbestos and asbestos-containing products and materials was agreed upon. An important part of this roadmap was ensuring labour market opportunities for workers who would likely be affected by changes in the market for asbestos-containing brake products. In 2002, the Australian Workplace Relations Ministers’ Council agreed to phase out all new chrysotile asbestos use by 2003 [26].

The complete asbestos ban enacted in 2003 was a significant victory for the trade union movement of Australia. This unfortunately represented a story of the lack of political will by governments at Federal and State levels to act in the health interests of their community. A key challenge for members of the AMWU was that although the manufacturing of asbestos-containing products had ceased, members were still exposed to asbestos in gaskets and asbestos friction products within existing and new machinery and plants. One particular dispute that occurred shortly after the 2003 ban highlighted the need for protection of both current and future workers from asbestos-containing machinery parts. Through the constant vigilance and efforts of trade unions, an agreement was reached that saw the removal of all the readily accessible asbestos-containing gaskets thereby removing the unnecessary risk to future employees who could have been exposed to asbestos.

## 4. More Recent Developments and Challenges

While 2003 marked an important milestone in actions to prevent asbestos-related disease in Australia, it was widely recognised that the long latency period between first asbestos exposure and asbestos-related disease meant that government agencies had to continue their response to this devastating disease.

### 4.1. Establishment of a National Asbestos Safety and Eradication Organisation

The Asbestos Safety and Eradication Agency of Australia (ASEA) was recently established to provide a national focus on asbestos issues which go beyond workplace safety to encompass environmental and public health issues. To the knowledge of the authors, no similar agencies at the national level exist in the world. ASEA has a broad set of functions that focus on encouraging, coordinating, monitoring and reporting on the implementation of the National Strategic Plan on Asbestos Awareness and Management 2014–2018 [27]. The National Strategic Plan provides a comprehensive and holistic framework for increasing awareness about the risks of asbestos exposure and managing asbestos containing materials (see Box 1). ASEA is a statutory authority established on 1 July 2013 following the enactment of the Asbestos Safety and Eradication Agency Act 2013. The operation of the Agency is the responsibility of the Commonwealth Minister for Employment. The Agency’s establishment was based on a 2010 summit jointly sponsored by the Cancer Council Australia, the Australian Manufacturing Workers’ Union, and the Australian Council of Trade Unions [28]. At this summit, the need for a national asbestos safety agency was highlighted as important due to poor community awareness about the risks of asbestos, disjointed approaches by various levels of government, and deficiencies in compliance with existing asbestos safety regulations.

Box 1 **Six key strategies of Australia’s National Strategic Plan on Asbestos Awareness and Management (Asbestos Safety and Eradication Agency, 2014, National Strategic Plan on Asbestos Awareness and Management 2014–2018).**

Strategy 1—Awareness: Increase public awareness of the health risks posed by working with or being exposed to asbestos.

Strategy 2—Best Practice: Identify and share best practice in asbestos management, education, transport, storage and disposal.

Strategy 3—Identification: Improve the identification and grading of asbestos and sharing of information regarding the location of ACMs.

Strategy 4—Removal: Identify priority areas where ACMs present a risk, identify the barriers to the safe removal of asbestos, and review management and removal infrastructure to estimate the capacity and rate for the safe removal of asbestos.

Strategy 5—Research: Commission, monitor and promote research into the prevention of asbestos exposure and asbestos-related disease.

Strategy 6—International Cooperation: Australia continues to play a leadership role in a global campaign for a worldwide ban on asbestos mining and manufacturing.

There is wide-held consensus that many houses in Australia built before 1990 have asbestos-containing materials. This has resulted in a high degree of occupational asbestos exposure for plumbers and electricians working in residential properties. In response, ASEA has worked with key trade groups to develop specific advice for these two groups of workers. The Agency has developed information aimed specifically at those working in the plumbing sector who have a very high chance of encountering asbestos in their day to day working lives [29]. This information was developed with the Plumbers and Pipe Trades Employees Union and Master Plumbers Australia. Similarly, electricians can be exposed to asbestos in a wide range of field specialties; from power stations to fixing up a cable in a street pit or conduit to a suburban home. Targeted information for electricians was developed by ASEA [30]. This information was developed in partnership with the National Electronical and Communications Association, the Electrical Trades Union, and Master Electricians Australia. This partnership approach with trade union and trade peak bodies is likely to increase the uptake of asbestos safety measures. Understanding the impact of the asbestos awareness campaigns in these specific sectors is an interesting public health evaluation activity in its own right.

Another key output recently produced by ASEA is Australia’s first National Asbestos Profile (NAP) [31]. National asbestos profiles assist countries in defining the baseline scenarios for asbestos consumption and asbestos-related disease, including population groups at most risk of asbestos exposure. Profiles also include information about enforceable asbestos exposure limits. Australia’s NAP draws on high-quality data to provide a history of asbestos consumption and regulation in Australia as well as more contemporary information about on-going asbestos issues in Australia, such as the importation of asbestos-containing materials from other countries. The NAP sits alongside the National Strategic Plan on Asbestos Awareness and Management 2014–2018 and will help in tracking over time the effectiveness of Australia’s asbestos safety efforts, including actions to eliminate asbestos-related disease.

Despite the excellent work of the agency, there are discussions at the time of writing this article within the public sphere of integrating the agency within a Commonwealth Government Department. However, in our view, this may detrimentally affect the ongoing need to have a high priority on actions and resources to prevent asbestos-related disease in Australia.

### 4.2. National Mesothelioma Surveillance Programs

Since the mid-1980s, Australia has made significant investment in collecting asbestos exposure data from people newly diagnosed with malignant mesothelioma. Three different iterations of this system were implemented—the Australian Mesothelioma Surveillance Program that operated between 1980 and 1985 [2], the Australia Mesothelioma Register (1986–2007) [3,18], and the recently re-established Australia Mesothelioma Registry operating since 2010 [32].

A national forum was held in 2009 to discuss options for Australian’s mesothelioma surveillance system, including collection of asbestos exposure data for people newly diagnosed with malignant mesothelioma. In July 2010, the Australian Mesothelioma Registry was reestablished as a consortium arrangement based at the Cancer Institute NSW with Safe Work Australia and academic, cancer registration, and other occupational health and safety groups [33]. The registry was fully in operation by 1 January 2011. The main aims of the Australian Mesothelioma Registry include to: (1) better understand the relationship between asbestos exposure and malignant mesothelioma; (2) identify the circumstances under which groups of individuals are exposed to potentially dangerous levels of asbestos and to facilitate prevention; and (3) assist the development of policies to best deal with the asbestos still present in our environment.

Detailed methods for the manner in which asbestos exposure data was assigned are published elsewhere [32,34,35]. Briefly, following the receipt of a confirmed malignant mesothelioma case, each cancer registry contacts the relevant clinician in order to obtain consent to contact the person with malignant mesothelioma. Consent is provided if the subject was diagnosed with malignant mesothelioma since 1 July 2010, is living at the time of the consent being given and is well enough to participate. Once clinician consent is granted, the person with malignant mesothelioma is contacted by the cancer registry to ask for his or her consent to participate in the collection of asbestos exposure data. Each consenting individual is provided with a postal questionnaire relating to his or her asbestos exposure history. Based upon data collected from the postal questionnaire, a tailored telephone interview is then conducted using a Web-based application called OccIDEAS [36,37]. All participants, regardless of their occupational history, are asked about asbestos exposure in occupational and non-occupational environments, including their home renovation activity. Exposure assessment algorithms available in OccIDEAS then determine the likelihood of asbestos exposure in occupational and non-occupational settings. Asbestos exposure data for people who developed malignant mesothelioma in Australia between 2010 and 2016 is detailed in Section 5.3 below. In terms of asbestos exposure in the wider context, van Oyen and colleagues estimated asbestos exposures for 537 combinations of 224 occupations and 60 industries for four time periods between 1943 and 2003. The highest average asbestos exposures were thought to have occurred in the shipyard, insulation and asbestos manufacturing industries. Forty-six combinations of occupation and industry categories were considered by the researchers to have had exposures exceeding the current Australian exposure standard (0.1 fibre/millilitre).

An ongoing challenge in the efforts by the public health and trade union movement in Australia in preventing asbestos-related disease has been attempts to close down the various iterations of the national mesothelioma surveillance program, including attempts to close the program and registry down in 1987, 1993 and 1995. There was strong lobbying from the trade union movement in 2007 to have the Australian Mesothelioma Registry re-established. The future of the Australian Mesothelioma Registry was again reviewed in 2016. Most recently, the Australian Mesothelioma Registry is in the process of being moved from the Cancer Institute NSW to the Australian Institute of Health and Welfare.

### 4.3. National Asbestos Safety and Awareness Campaigns

In recognising that the Australia community will continue to be at risk from asbestos-related disease through occupational and non-occupational asbestos exposure, a public asbestos awareness campaign was set up. The Asbestos Awareness Campaign and the asbestosawareness.com.au website was launched in 2011. It is the initiative of the Heads of Asbestos Coordination Authorities’ Asbestos Education Committee, consisting of a number of key stakeholders including the not-for-profit, corporate, government (i.e., SafeWork NSW) and asbestos-related diseases support groups and research organisations. Since launching the campaign in New South Wales in 2012, the campaign was rolled out nationally and works closely with community stakeholders including local and state governments as well as businesses, leveraging the media to increase awareness of the dangers of asbestos and how to manage it safely. The national Asbestos Awareness Campaign has been highly successful and has won a number of international high-profile communications awards.

A flagship asbestos awareness campaign in Australia is the Betty House. Betty is designed as a mobile home unit that can be driven around different locations to engage the general public about where asbestos can be found in Australian homes that are built or renovated before 1987. On the outside, the Betty House is similar in appearance to a typical Australian home built with asbestos-containing materials. Inside, Betty provides audiovisual explanations of asbestos-containing materials and products in the main parts of a house including the bathroom, kitchen, and living room. Importantly, when the Betty House travels around different areas, asbestos awareness campaigns are also simultaneously timed to occur in local media and in partnership with local governments in that area. Betty has travelled extensively across Australia in conjunction with widespread media campaigns driving traffic to Betty’s Facebook page and the AsbestosAwareness.com.au website. The website provides a plethora of information on the dangers of asbestos and how to manage it in various circumstances (Table 1).

An area of potential further public health research is better understanding of the impact of the national asbestos awareness campaign and whether there has been a sustained behaviour change of actions to prevent asbestos-related disease among the campaign’s target audience.

## 5. Asbestos-Related Disease in Australia

Despite a complete asbestos ban being in place since 2003, malignant and non-malignant asbestos-related diseases (ARDs) continue to be diagnosed in Australia. While the incidence of malignant mesothelioma appears to be stabilising, if not slowing, there were still 700 people newly diagnosed with malignant mesothelioma in 2016 [32]. Tracking malignant and non-malignant ARDs will help inform researchers and policy makers of the effectiveness of implemented asbestos bans and of ongoing or new occupational or non-occupational asbestos exposure risks.

### 5.1. Descriptive Epidemiology of Malignant Mesothelioma

**Figure 1:**
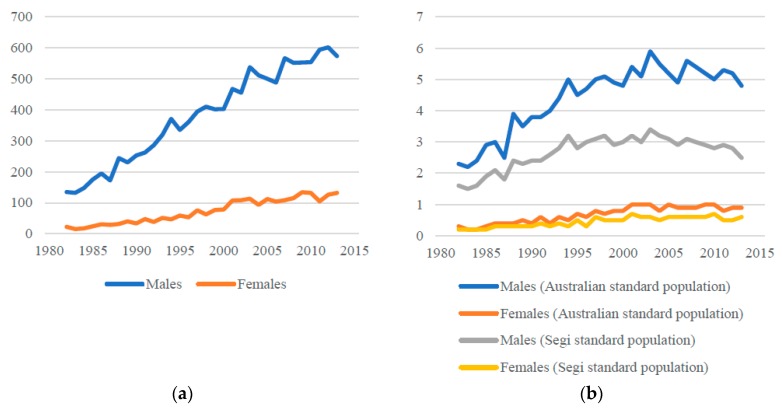
Incidence of malignant mesothelioma, Australia, 1982–2013, using data published by the Australian Institute of Health and Welfare. (**a**) Incident cases of malignant mesothelioma; (**b**) Age-standardised incidence rates per 100,000 of malignant mesothelioma using Australian and Segi world standard populations.

Asbestos use in Australia has resulted in a significant ARD burden including malignant and non-malignant disease. The most reliable data on Australia’s ARD burden is from cancer registry data since there are legal requirements to report incident cancer cases [38]. Using data from the Australian Institute of Health and Welfare and the Australian Mesothelioma Registry [32,39], 16,679 people were newly diagnosed with malignant mesothelioma in Australia between 1982 and 2016 with 84% (13,928) of those incident cases occurring in men (Figure 1a). The majority of incident cases (around 70%) during this period were aged 65 years or more.

It is worth observing that Australia’s asbestos consumption peaked in the 1970s, with around 700,000 tonnes of asbestos consumed during this decade. It is possible to correlate asbestos consumption at the population level in previous decades to more current occurrence of asbestos-related disease. In Australia, around 100 tonnes of asbestos consumed has led to around one death due to malignant mesothelioma. Further research is required to collate, estimate and critique the correlations shown by various researchers between previous asbestos consumption and the burden of asbestos-related disease that occurs decades later.

Age-standardised malignant mesothelioma incidence rates per 100,000 for males and females combined were highest in the 2000s. For example using the Australian standard age population, the combined male and female incidence rate per 100,000 in 2003 was 3.2 and has declined since then (2.5 per 100,000 in 2016). The highest male malignant incidence rate, also in 2003, was a rate of 5.9 per 100,000 using the Australian standard age population (Figure 1b). Like many other countries, female malignant mesothelioma incidence rates are substantially lower than male rates. Since 1997, the age-standardised incidence rate for women diagnosed with malignant mesothelioma in Australia has ranged between 0.8–1.0 per 100,000 (Australian standard population) and 0.5–0.7 per 100,000 (Segi world population) (Figure 1b). Given the rapid mortality between a malignant mesothelioma diagnosis and death, mortality rates are close in value to the incidence rates.

### 5.2. Estimates of Australia’s Non-Malignant Asbestos-Related Disease Burden

Asbestos-related diseases also include non-malignant diseases such as asbestosis. Australian data on non-malignant ARDs need to be interpreted with some caution as legal disease registry reporting requirements differ from that for malignant diseases. However, a view of Australian hospitalisation data is insightful. These hospitalisation data reflect the number of separate hospital episodes rather than the number of people hospitalised [40].

**Figure 2:**
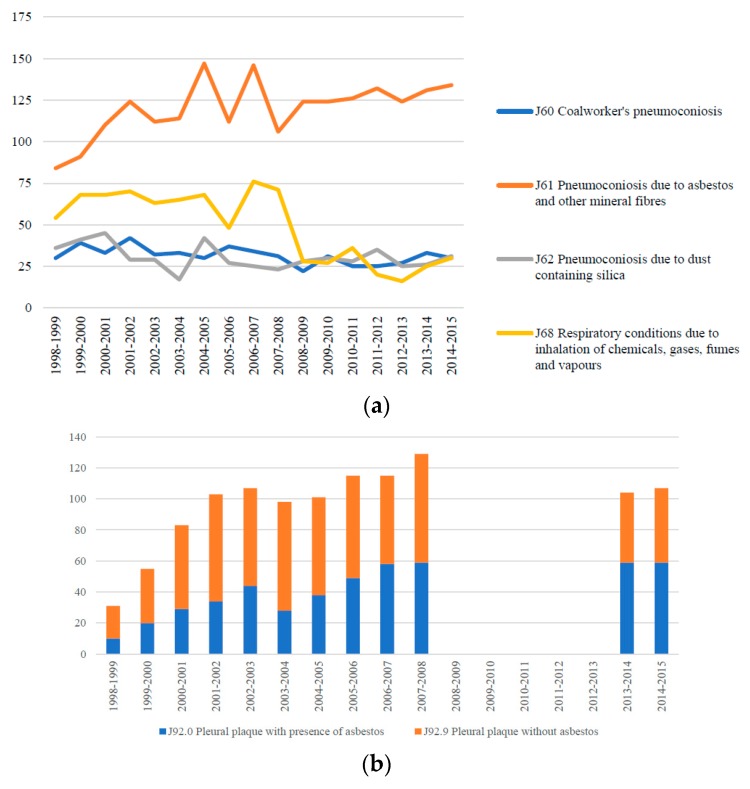
(**a**) Number of hospital separations, Australia, 1998–1999 through to 2014–2015, for a primary diagnosis of respiratory diseases due to external agents; (**b**) Number of pleural plaque hospital separations (ICD code J92), Australia, 1998–1999 through to 2014–2015, by whether asbestos was present or not.

Between July 1998 and June 2015, there were 2041 hospitalisations for asbestosis (ICD-10 code J61). This can be compared to 833 hospitalisations during the same period for respiratory conditions due to inhalation of chemicals, gases, fumes and vapours (ICD-10 code J68) and 517 hospitalisations for silicosis (ICD-10 code J62) (Figure 2a). Hospitalisation data is also collected and reported for pleural plaques with and without the presence of asbestos (ICD code J92.0 and J92.9). National hospitalisation data suggest that there were 1148 hospital episodes for pleural plaques between July 1998 and June 2015 (Figure 2b). Data were not available from July 2008 through to June 2013. Nevertheless of all pleural plaques hospitalisations reported between 1998 and 2015, 43% (487 hospitalisations) were recorded as pleural plaques with the presence of asbestos.

### 5.3. Asbestos Exposure Data

In the Australian Mesothelioma Registry, each person who had asbestos exposure data collected is assigned as having probable, possible, or unlikely asbestos exposure above background exposure levels, with probable exposure further defined into high, medium, and low likelihoods for asbestos exposure. The assessment of asbestos exposure does not account for duration, frequency, or intensity of asbestos exposure. A sequential process is used such that occupational asbestos exposure is first assessed. If it is considered that a subject’s occupational asbestos exposure was unlikely, questions regarding that person’s non-occupational asbestos exposure are then initiated. If an individual completed a number of job-specific questions in the same field, then the highest probability of exposure is recorded.

**Figure 3:**
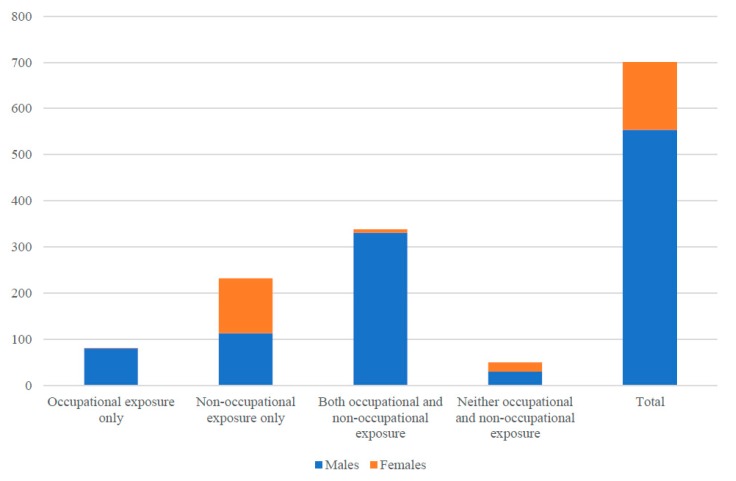
Number of people recorded as being exposed to asbestos through occupational and non-occupational in settings in Australia, 1 July 2010 through to April 2017, using data reported the Australian Mesothelioma Registry.

The most recent report from the Australian Mesothelioma Registry presents data on the number of people recorded as having occupational or non-occupational exposure [32]. Asbestos exposure data from the Australian Mesothelioma Registry is available for 701 people whose asbestos exposure data was collected between 1 July 2010 and April 2017 (Figure 3). Women made up around 20% of all exposure data collected with some clear patterns for males and females according to the type of asbestos exposure. Notably, an almost equal proportion of males (113, 48%) and females (119, 52%) were assigned as having non-occupational asbestos exposure. This is in contrast to 1% of females (1 out of 82 cases) who were assigned as having occupational asbestos exposure. Fifty people were assigned as having no evidence of occupational or non-occupational asbestos exposure.

## 6. Conclusions

It is widely recognised that the devastating burden of malignant and non-malignant asbestos-related disease is preventable. One of the main actions that countries can take to prevent asbestos-related disease is to implement bans on the use of asbestos and asbestos-containing products (Box 2).

Box 2 **Key lessons learnt applicable to other countries to assist in the elimination of asbestos and asbestos-related diseases.**

- Working across different government, non-government and private sector agencies is vital to ensure a comprehensive understanding of asbestos consumption volumes and patterns in a country including the import and export of asbestos or asbestos-containing materials and products. These data can be directly used in the development of the national asbestos profile.
- Working with the health and workers’ compensation agencies will help to determine what, if any, qualitative or quantitative data are available on the incidence of malignant and non-malignant asbestos-related diseases in the country.
- It is important to bring various government, non-government and industry sector groups to the table to discuss the health, social and employment impacts of the introduction of an asbestos ban.
- Implementing a ban on the import or export of asbestos or asbestos-containing products will not lead to an immediate impact on the incidence of asbestos-related disease, these effects may take decades to become apparent.

Even once asbestos has been banned, many years of public health preventative action is required. This includes safely handling or eradicating asbestos and asbestos-containing materials from occupational and non-occupational environments to avoid exposure to asbestos in its breathable form. However, neither the task of banning of asbestos nor the community prevention actions required to prevent epidemics of asbestos-related disease are easy to implement. Ensuring that governments take timely policy and regulatory decisions to implement asbestos bans often involves continuous and sustained advocacy efforts from the non-government sector, including the trade union movement. In Australia, the trade union movement was critical in its role of forcing government action to ban asbestos, particularly the banning of chrysotile in the early 2000s. Asbestos-related disease surveillance is also a critical input for advocating healthy public policy as well as for implementing asbestos awareness and safety campaigns targeting specific subgroups of the population. For example, tradespeople who have contact with asbestos-containing products in the residential or commercial built environment as well as the Australian community who live in or are exposed to respirable asbestos fibres during home renovation.

Understanding the pathway to Australia’s asbestos ban is critical for the many countries continuing to use asbestos. However, it is more important to realise that this is only part of an unfinished story. A complete asbestos ban was in place in 2003. Almost fifteen years later, Australia is only now seeing the peak of its asbestos-related disease epidemic with ongoing risks of asbestos exposure. The Australian community needs to remain vigilant to the public health risk of asbestos exposure from existing asbestos or asbestos-containing materials as well as exposure to asbestos-containing materials that are brought into Australia despite regulations being in place. As public health researchers and advocates, we have an obligation to continue to monitor and understand the patterns of Australia’s asbestos-related disease epidemic through high-quality disease surveillance and epidemiological research to determine the effectiveness of public health prevention efforts.

## Figures and Tables

**Table 1:** On-line guides, fact sheets, handbooks, videos and database (Australian asbestos awareness resources available from the website AsbestosAwareness.com.au).

AsbestosAwareness.com.au	On-line Guides, Fact Sheets, Handbooks, Videos and Database
Asbestos in the Home Information for do-it-yourself home renovators about the potential presence of asbestos, asbestos exposure during renovations and what not to do.	20 Point Safety Check Healthy House Checklist—A Homeowner’s Guide To Identifying Asbestos-Containing Material To Manage It Safely Where asbestos may be found—an Interactive House diagram Working safely with asbestos around the home Safe practices for homeowners repairing or removing small amounts of asbestos materials Safe practices for rural & regional homeowners & farmers repairing or removing small amounts of asbestos materials Video—Asbestos in your home: The Ultimate Renovator’s Guide
Asbestos for Tradies Guidelines and checklists to assist tradespersons and those working on residential properties to manage asbestos safely to minimise the risks to their health, their colleagues, families and bystanders.	20 point Safety Check List for Tradies Working on Residential Properties A Tradie’s Guide to Safe Practices in Managing Asbestos in Residential Properties A Tradesperson’s Guide to Asbestos Containing Material in Domestic Properties Residential Asbestos Checklist—for 23 different trades Asbestos Personal Protection Equipment (PPE) for tradies
Commercial Properties	The Asbestos Management Handbook for Commercial and Non-Residential assists property owners, managers, contractors, project managers and foremen to develop and maintain effective asbestos management plans and procedures in the workplace.
Naturally Occurring Asbestos (NOA)	The Naturally Occurring Asbestos Management Plan assists landowners to understand NOA and manage it in accordance with regulations.
Asbestos Product Database	Asbestos containing products used both domestically and industrially are described with accompanying photos, product descriptions and application.
